# Glucose Metabolism Regulates T Cell Activation, Differentiation, and Functions

**DOI:** 10.3389/fimmu.2015.00001

**Published:** 2015-01-22

**Authors:** Clovis S. Palmer, Matias Ostrowski, Brad Balderson, Nicole Christian, Suzanne M. Crowe

**Affiliations:** ^1^Centre for Biomedical Research, Burnet Institute, Melbourne, VIC, Australia; ^2^Instituto de Investigaciones Biomédicas en Retrovirus y SIDA, Facultad de Medicina, Universidad de Buenos Aires, Buenos Aires, Argentina; ^3^Department of Microbiology, The University of the West Indies, Kingston, Jamaica; ^4^Department of Infectious Diseases, Monash University, Melbourne, VIC, Australia; ^5^Infectious Diseases Department, The Alfred Hospital, Melbourne, VIC, Australia

**Keywords:** glucose transporter 1, PI3K, metabolism, mTOR, HIF-1α, immune activation, HIV, inflammation

## Abstract

The adaptive immune system is equipped to eliminate both tumors and pathogenic microorganisms. It requires a series of complex and coordinated signals to drive the activation, proliferation, and differentiation of appropriate T cell subsets. It is now established that changes in cellular activation are coupled to profound changes in cellular metabolism. In addition, emerging evidence now suggest that specific metabolic alterations associated with distinct T cell subsets may be ancillary to their differentiation and influential in their immune functions. The “Warburg effect” originally used to describe a phenomenon in which most cancer cells relied on aerobic glycolysis for their growth is a key process that sustain T cell activation and differentiation. Here, we review how different aspects of metabolism in T cells influence their functions, focusing on the emerging role of key regulators of glucose metabolism such as HIF-1α. A thorough understanding of the role of metabolism in T cell function could provide insights into mechanisms involved in inflammatory-mediated conditions, with the potential for developing novel therapeutic approaches to treat these diseases.

## Introduction

The immune system comprises specialized cell populations that are conditioned to respond rapidly and vigorously to antigenic and inflammatory signals. Most research has focused on these signals in guiding immune responses. Now emerging data indicate that cellular metabolism regulates immune cell functions and differentiation, and consequently influences the final outcome of the adaptive and innate immune response (1–4). The growth, function, survival, and differentiation of an activated immune cells depend on dramatic increases in glucose metabolism as fuel, a process that is directly regulated and has a profound impact on health and disease (1, 5–7).

Inflammatory conditions such as HIV infection results in a heightened inflammatory state that affects the availability and use of energy. This in turn influences T cell activation and functions (8, 9). Identifying the pathways that coordinate the metabolic processes during inflammatory conditions, as observed in HIV infection will potentially provide new therapeutic opportunities.

## Metabolic Profiles of Immune Cells during Immune Activation

The functions of peripheral T cells are maintained and are intimately linked to metabolism. Specific effector functions are unable to proceed without the cell adopting the appropriate metabolic state (10, 11). Research into T cell metabolism has provided valuable insight into the pathways that are important for T cell fate, plasticity, and effector functions. T cells rapidly transition between resting catabolic states (naïve and memory T cells) to one of growth and proliferation (effector T cells) during normal immune responses (10, 11).

The commitment of an immune cell to a specific metabolic pathway depends on the particular function. This is evident in the subsets of CD4+ T cells where effector T cells and Th17 cells rely on aerobic glycolysis, while memory T cells and T regulatory cells (Treg) rely on fatty acid oxidation to produce energy (12). Aerobic glycolysis is also utilized for energy by activated dendritic cells, neutrophils, and pro-inflammatory macrophages (13).

The vast majority of evidence supporting the significance of metabolism in immune cell functions is derived mainly from *in vitro* and animal models. The reasons why T cells adopt specific metabolic programs and the impact this has on their function in the context of human diseases such as HIV infection remains unclear.

## How is Glucose Used by Immune Cells to Produce Energy?

Glucose is transported into T cells via the high affinity Glucose transporter 1 (Glut1), which is the major glucose transporter on T cells (14, 15). Through a rate limiting step catalyzed by hexokinase, glucose is trapped inside the cells where it is metabolized via glycolysis. During this process, each glucose molecules is broken down into pyruvate with a net production of two ATP molecules. Most non-proliferating and terminally differentiated T cells such as naïve and memory T cells completely oxidize pyruvate via the tricarboxylic acid (TCA) cycle to generate NADH and FADH2 that fuel oxidative phosphorylation producing 36 molecules of ATP per glucose molecule. When T cells are activated, pyruvate is transformed into lactate regenerating NAD+ that subsequently engages glycolytic reactions.

It may seem counterintuitive that T cells, which have increased demand for energy would be involved in exploiting a relatively insufficient process to generate energy. Whilst glycolysis is less efficient in generating ATP than oxidative phosphorylation, it is a rapid process occurring independently of mitochondrial function. Furthermore, a widely held assumption is that the shift from oxidative phosphorylation to increased aerobic glycolysis by rapidly proliferating T cells diverts the use of glucose for macromolecular biosynthesis (16).

## Glucose Metabolism in Naïve and Activated T Cells

Upon maturation in the thymus, naive CD4+ T cells recirculate between the blood and secondary lymphoid organs. The immune quiescence of naïve T cells is accompanied by a catabolic metabolism, characterized by the breakdown of glucose, fatty acids, and amino acids to generate intermediate metabolites, which enter the mitochondrial TCA cycle (17). The interconversion of metabolites in the TCA cycle generates energy and reducing equivalents, which subsequently enter the oxidative phosphorylation pathway effectively increasing ATP production.

The quiescence of naïve T cells is interrupted upon engagement of the T Cell Receptor (TCR) by a specific antigen/MHC class II complex displayed on the surface of dendritic cells, concurrently with the recognition of costimulatory molecules by the receptor CD28. These two signals trigger T cell activation, the secretion of IL-2, cellular proliferation referred to as clonal expansion, and their differentiation into an effector phenotype. These changes in the activation status of CD4+ T lymphocytes not only require energy, but also increased demand for metabolic precursors for the biosynthesis of proteins, nucleic acids, and lipids to fuel clonal expansion and subsequent differentiation into effector cells. Therefore, efficient T cell activation requires profound changes in cellular metabolism (18, 19). In effect, energy generation through the TCA cycle and oxidative phosphorylation is interrupted and have been thought to be replaced by glycolysis, in which glucose is converted to lactate in the cytosol, even when sufficient oxygen is available to perform oxidative phosphorylation (5, 20).

The peculiar promotion of glycolysis in the presence of normal oxygen levels is referred to as aerobic glycolysis and it is also a hallmark of cancer metabolism (21, 22). Although less efficient in terms of energy production, aerobic glycolysis generates metabolic intermediates that are used in anabolic pathways required to sustain cell growth and to produce daughter cells. However, more recently the dogma that CD4+ T cells simply switch from an oxidative to glycolytic metabolism has been challenged. Cao and colleagues demonstrated that oxidative phosphorylation is strongly induced during CD4+ T cells activation (23). By comparing CD4+ and CD8+ T cells, the researchers showed that these cells utilize distinct metabolic strategies to meet their functional demands. Following activation, CD8+ T cells had a higher glycolytic flux than CD4+ T cells. On the other hand, CD4+ T cells also induced glycolysis upon activation, but had greater mitochondrial content and oxidative metabolism than CD8+ T cells. Nevertheless their observation that glycolytic inhibition by 2 deoxy-glucose (2-DG) suppressed CD4+ T cell growth, and that rotenone inhibited both CD4+ and CD8+ T cell proliferation underscores the significance of glycolysis and oxidative metabolism in T cell activation (23). It is therefore apparent that T cell activation is not accompanied merely by a switch from oxidative metabolism to glycolysis, but that both pathways are upregulated to support bioenergetic demands. This intimate interrelationship between T cell activation and metabolism led to the concept that changes in T cell metabolism are not simply a consequence of antigen-induced activation, but rather a parameter that determines T cell proliferation and fate decisions (5, 24).

## Signaling Pathways Regulating Glucose Metabolism in T Cells

**Figure 1:**
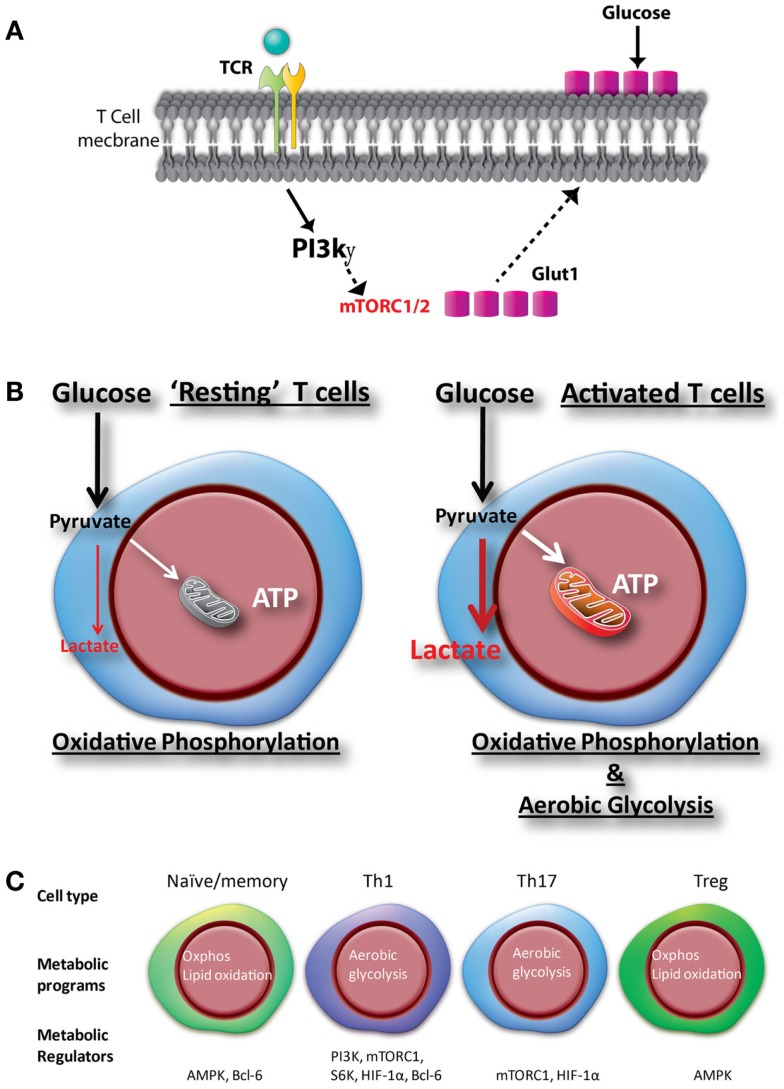
**Glucose metabolic programs regulate glucose uptake, activation, and differentiation of CD4+ T cells**. **(A)** Mitogenic stimulation or engagement of the TCR complex activates the phosphoinositide 3-kinase (PI3K)-gamma (PI3Kg) subunit in CD4+ T cells. Activation of the PI3Kγ in CD4+ T cells promote Glut1 trafficking from the cytoplasm to the cell membrane and increases glucose uptake to sustain activation. **(B)** Upon activation, T cells increase glucose uptake through Glut1, which facilitate increased oxidative phosphorylation and glycolysis to sustain cell growth and proliferation. **(C)** Unique metabolic changes upon differentiation of T cells toward different subsets, regulated by various transcription factors and signaling pathways.

In activated T cells, the rapid induction of glycolysis is promoted by the increase in the activity of several enzymes and proteins, which are regulated at the transcriptional and posttranscriptional levels. Following T cell activation, Glut1, is translocated to the surface of CD4+ T cells (25–28). This occurs in response to the activation of the phosphoinositol-3 kinase (PI3K)-Akt pathway that triggers the recruitment of Glut1 from the cytoplasmic pool to the cell surface. Increased Glut1 expression and glucose uptake by activated T cells is accompanied by increased glycolysis (Figures 1A,B) (29). An abnormal transduction of Akt signaling was discovered among Fas-associated protein with death domain (FADD) knock out thymocytes, and was partly responsible for a decline in Glut1 expression, a corresponding decrease in glucose uptake, increased apoptosis, and reduced cell numbers (30). In addition, T cell activation accompanies induction of the mammalian target of rapamycin (mTOR) pathway. mTOR is a serine/threonine kinase that forms two multiprotein complexes, mTORC1 and mTORC2, as determined by the association with different adapter and scaffolding proteins. mTOR activation regulates a myriad of cellular functions, including growth, apoptosis, differentiation, and metabolism (31, 32). Recently, the mTOR pathway has generated enormous attention due to the regulation and differentiation of distinct T cell subsets by different mTORC complexes (7), and the considerable interest in these complexes by the pharmaceutical industry (33). Other signaling pathways that have been associated with glucose metabolism in T cells are the extracellular signal-related kinase (ERK) (34), signal transducer and activator of transcription (STAT5) (15), some MAPKinases (35), and hexokinase II (36). However, the magnitude by which these pathways regulate T cell metabolism may vary depending on the precise environmental conditions. Indeed, it is also likely that these pathways may also co-operate with the PI3K-Akt and mTOR pathways to regulate metabolic reprograming of T cells.

## mTOR Regulation of Glucose Metabolism, T Cell Activation, and Differentiation

During metabolism in CD4+ T cells, activation of the mTOR by TCR/CD28 coligation interrupts catabolic metabolism by regulating fatty acid oxidation and oxidative phosphorylation (12). Concurrently, mTOR induces the transcription of many key glycolytic enzymes (37). Thus, the increase in glucose uptake due to Glut1 translocation, together with enhanced transcription of glycolytic enzymes results in an important increase in the glycolytic flux. The regulation of metabolic pathways by mTOR is mainly achieved through the activation of downstream transcriptional factors, such as Bcl-6 involved in the regulation of T cell immune function (38, 39), which reinforces the current paradigm that metabolism and T cell function are deeply interconnected. In particular, mTOR activation stimulates the activity of the transcription factor Myc, which plays a key role in the metabolic switch following activation by promoting expression of enzymes involved in aerobic glycolysis and other anabolic pathways (17). In addition, mTOR enhances the translation of the mRNA encoding HIF-1α. The increase in HIF-1α level also facilitates the expression of critical components of the glycolytic pathway and regulates the balance between Treg and inflammatory Th17 differentiation through direct transcriptional activation of RORγt thus promoting T cell differentiation and activation (40, 41).

## Metabolic Signatures of CD4+ T Cell Lineages

The development of an effective and balanced immune response is largely determined by the differentiation status (Naïve/memory) and effector profile of T cells (Th1, Th17, and Treg), and is controlled by distinct metabolic programs (Figure 1C). Each of these lineages has a distinctive functional property, largely determined by the production of a defined subset of cytokines (42). The lineage commitment of activated T cells is determined by the integration of multiple cues present in the immune microenvironment at the moment of activation. Interestingly, both the metabolic and immunologic programs are coordinated by the mTOR (43). Thus, mTOR activity is required for the differentiation of all the CD4+ T cell effector subsets but not for the differentiation of Tregs (44, 45). Indeed, whereas effector T cells are highly glycolytic, Tregs have a metabolism dominated by the oxidation of fatty acids followed by oxidative phosphorylation (12).

The activity of mTOR not only influences effector versus regulatory decisions, but also plays a critical role in the differentiation of the different effector T cell profiles. As follows, signaling from the mTORC1 is required for the differentiation of Th1 and Th17 but not of Th2 cells (46). Conversely, the mTOR signaling through mTORC2 is required for the differentiation of Th2 cells (46).

### Metabolic programing of Th1 cells

Th1 cells are functionally characterized by the production of IFNγ and TNF, which are of utmost importance in the induction of cell-mediated immunity against obligate intracellular pathogens, such as viruses, as well as bacteria, such as *M. tuberculosis*. Th1 cells possess a high glycolytic rate, which is paralleled by high surface expression levels of Glut1 (12). Remarkably, the glycolytic metabolism of Th1 cells dramatically influences their functionality, as evidenced by the fact that inhibition of glycolysis severely suppresses the secretion of IFNγ (6, 10). In addition to the role of aerobic glycolysis in biomass production, glycolysis and IFNγ production present one extra level of interaction. Indeed, it has been proposed that glycolytic enzymes can regulate the effector phenotype of T cells by performing non-metabolic functions. For example, the glycolytic enzyme GAPDH, if not engaged in glycolysis, can bind IFNγ mRNA post-transcriptionally, blocking the translation of this cytokine. Thus, aerobic glycolysis would be required to engage GAPDH in its metabolic functions, liberating the IFNγ mRNA for translation, thereby allowing these cells to attain full effector functions (10). Interestingly, studies conducted by Cham and Gajewski elegantly demonstrated that IFNγ, but not IL-2, production is preferentially inhibited by limiting glucose conditions (47). This further highlights the important link between glucose metabolism regulated by mTORC1, and CD8+ T cell effector functions. Although inhibition correlated with reduced phosphorylation of p70S6 kinase and eIF4E binding protein 1, surprisingly, inhibition of mTOR failed to block T cell cytokine production under their experimental conditions (47). This illustrates the complex relationship between transcriptional and post transcriptional control of T cell effector functions, mediated by metabolic reprograming.

### Metabolic programing of Th17 cells

Th17 cells produce IL-17, IL-21, and IL-22 and are critical for the control of extracellular bacteria and mucosal immunity. Moreover, Th17 cells are important for of a number of autoimmune processes (48). Like Th1 cells, Th17 cells are highly glycolytic (12). In addition to the requirement of signaling from the mTORC1 (46), Th17 cell differentiation is critically dependent on the transcription factor HIF-1α. HIF-1α not only stimulates the glycolytic activity of Th17 cells, but also transcriptionally activates the master transcription factor RORγt (40), which subsequently directs the differentiation program of Th17 cells (49). Thus, it is therefore clear that metabolic programs can now be used to identify and classify effector T cell lineages as reviewed by Maclver and colleagues (7).

## Metabolic Programs in Differentiation and Expansion of Tregs

Lipid oxidation via AMPK and oxidative phosphorylation are considered the predominant metabolic programs in differentiated Tregs (7, 12). However, Neildez-Nguyen and colleagues have recently shown that higher expression of Glut1 is detected on mouse Tregs generated under hypoxic (5% O_2_) culture conditions compared to those cultured under ambient oxygen levels (21% O_2_) (50). Indeed following differentiation, amplification of the committed Tregs was explicitly favored by low oxygenation, and by glycolysis probably through induction of Glut1 on the cell membrane (50). This observation underscores the significance of culture conditions such as oxygen levels in regulating metabolism and thus cautions how one interprets and relate *in vitro* metabolic activity to those in humans. The significance of these distinctions is confirmed by the expression and stability of HIF-1α, which is highly dependent on the level of oxygenation in the cellular environment. Augmented Glut1 expression, mediated by HIF-1α, is observed in response to low O_2_ levels in several cell types including CD4+ T cell (9). HIF-1α transcriptionally activates genes encoding glucose transporters, and rate limiting enzymes involved in glycolysis, and therefore plays a significant role in T cell differentiation and functions (39, 51).

## T Cell Metabolism in Inflammatory Diseases

Despite the overwhelming evidence suggesting that specific metabolic alterations is associated with T cell functions and differentiation; how these metabolic changes influences immune functions in human diseases has only recently been examined. Studying Glut1 levels on immune cells, Palmer and co-workers have shown that increased glycolytic metabolism in CD4+ T cells is associated with abnormally high levels of immune activation, and low CD4+ T cell count in HIV-infected persons (52), at least in part due to “metabolic exhaustion” of these cells. Indeed, recent investigations have demonstrated that Glut1 is a CD4+ T cell activation marker essential for cell growth and proliferation, and HIV infection *in vitro* (9, 53). It is unknown whether other inflammatory conditions such as obesity, diabetes, cardiovascular diseases, and rheumatoid arthritis can impact cellular metabolism of T cells and other immune cells. In the context of HIV infection, an established chronic inflammatory disease, increased glucose metabolism in inflammatory monocyte subsets was associated with elevated levels of markers of inflammation (54, 55).

As discussed above, following activation and differentiation, the pro-inflammatory CD4+ T cell subsets are distinguished from the anti-inflammatory CD4+ Tregs based on their metabolic signatures. Thus, intense investigations are now focused on the hypothesis that elevated glycolysis is a hallmark of inflammatory cells. Indeed, inhibition of glycolysis by rapamycin has been shown to facilitate the generation of murine naturally occurring CD4+CD25+Foxp3+ Tregs *in vitro*, which were able to prevent allograft rejection *in vivo* (56), illustrating the link between T cell differentiation, metabolism, and immunity.

Recently, the role of oxidative stress, a hallmark in several inflammatory conditions has been discussed in the framework of metabolism in immune cells (1). In inflammatory macrophages, reactive oxygen species (ROS) have been implicated in increased Glut1 expression and glycolysis, mediated in part by NF-kB signaling (1). Data regarding the role of ROS in T cell metabolism are sparse; however, upon T cell activation, mitochondrial ROS are generated within minutes, and at low levels is associated with cellular proliferation (57, 58). Therefore, a plausible model in the context of T cells is that ROS induce HIF-1α by activating PI3K/mTOR and or NF-kB-linked signaling to upregulate metabolic pathways that facilitate T cell expansion and proliferation (59). Another important consideration is the interaction between T cells and other immune cells such as monocytes and macrophages. Inflammatory mediators produced by activated monocytes and macrophages are potential sources of activating stimuli for T cells (54), thus a thorough understanding of the shared metabolic checkpoints by which diverse inflammatory cues and oxidative stress modulate metabolic programing will provide important insight into combined approaches to target cellular metabolism in T cells. Moreover, the prominence of immune cells in controlling inflammatory-associated inflammation such as obesity has now gained considerable attention (60). The exciting advances in immunometabolism may provide new opportunities to develop novel interventions for the treatment of inflammatory and metabolic diseases.

## Conflict of Interest Statement

The authors declare that the research was conducted in the absence of any commercial or financial relationships that could be construed as a potential conflict of interest.
